# Dietary Supplementation of Enzymatically Treated *Artemisia annua* L. Improves Lactation Performance, Alleviates Inflammatory Response of Sows Reared Under Heat Stress, and Promotes Gut Development in Preweaning Offspring

**DOI:** 10.3389/fvets.2022.843673

**Published:** 2022-03-25

**Authors:** Liang Xiong, WenFei Zhang, Hao Zhao, ZheZhe Tian, Man Ren, Fang Chen, WuTai Guan, ShiHai Zhang

**Affiliations:** ^1^Guangdong Provincial Key Laboratory of Animal Nutrition Control, College of Animal Science, South China Agricultural University, Guangzhou, China; ^2^College of Animal Science, Anhui Science and Technology University; Anhui Provincial Key Laboratory of Animal Nutritional Regulation and Health, Fengyang, China; ^3^College of Animal Science and National Engineering Research Center for Breeding Swine Industry, South China Agricultural University, Guangzhou, China

**Keywords:** *Artemisia annua* L., heat stress, hormone level, inflammatory factor, intestinal morphology

## Abstract

*Artemisia annua* L., which is known for its antimalarial compound artemisinin, has commonly been used for its anti-inflammatory and antibacterial functions. Enzymatically treating *Artemisia annua* L. can improve its bioavailability. The purpose of this study was to investigate the effects of dietary enzymatically treated *Artemisia annua* L. (EA) supplementation in late gestation and lactation diets on sow performance, serum hormone, inflammatory cytokines, and immunoglobulin level of heat-stressed sows. A total of 135 multiparous sows (Large White × Landrace) on day 85 of gestation were selected and randomly distributed into 3 groups with 45 replicates per group. The control group was reared under standard conditions (temperature: 27.12 ± 0.18°C, THI (temperature-humidity index): 70.90 ± 0.80) and fed with basal diet. The heat stress (HS) and HS + EA groups were raised in heat-stressed conditions (temperature: 30.11 ± 0.16°C, THI: 72.70 ± 0.60) and fed with basal diets supplemented with 0 or 1.0 g/kg EA, respectively. This trial lasted for 50 consecutive days until day 21 of lactation. Compared with the control group, HS increased the concentrations of serum endotoxin and heat shock protein 70 (HSP-70), and inflammatory cytokines in serum, colostrum, and 14 day milk of sows. Meanwhile, the EA supplementation decreased levels of serum endotoxin, HSP-70, and inflammatory cytokines in both sows and offspring and increased serum triiodothyronine (T_3_) level and average daily feed intake (ADFI) of sows. In addition, EA significantly improved average daily gain (ADG) and altered intestinal morphology with an increased villus height in the duodenum and ileum of piglets. Collectively, EA supplementation at 1.0 g/kg in late gestation and lactation diets alleviated the adverse effects of HS, which were reflected by enhancing ADFI and decreasing endotoxin as well as inflammatory cytokine levels in the serum and colostrum of heat-stressed sows, while promoting ADG and gut development of their offspring.

## Introduction

Heat stress (HS) has been reported to seriously affect the production performance (1), immune status (2), plasma hormone profiles (3), intestinal integrity and function (4), oxidative stress (5), and inflammation and heat shock response (6) of livestock, and is estimated to lose over US $450 million annually (7). In addition, global warming worsens the already serious problem of HS for the pig industry in tropical and subtropical regions around the world (8). Due to a lack of functional sweat glands and the existence of a thicker layer of subcutaneous adipose tissue that impedes radiant heat loss, sows are more susceptible to HS (9). HS normally reduces appetite and feed intake in sows which results in a negative energy balance and nutrient supply deficit for sows and impairs lactation performance further (9). Many detrimental effects of HS can be attributed to HS-induced intestinal barrier dysfunction (10). HS exposure directly impairs gut integrity and increases endotoxin concentration, which causes a severe immune inflammatory response and cytokine-induced febrile response in sows (11). The long-term effects of maternal HS exposure might induce intestinal dysfunction of offspring, especially gut permeability and barrier function (12).

Currently, supplementing animal diets with natural phytogenic additives, which can alleviate HS, have received extensive attention in consideration of the safety of animal-origin food (13–15). These extracts can be used as anti-inflammatory agents due to the ability of scavenging free radicals or alleviating inflammatory response (16, 17). *Artemisia annua* L. (*A. annua*) is a natural herb belonging to the genus artemisia of Compositae, famous for its antimalarial compound artemisinin (Qinghaosu) (18). Approximately 600 secondary metabolites have been identified and separated in *A. annua*, including sesquiterpenes, monoterpenes, flavonoids, and phenolic compounds. Among these compounds, artemisinin characterizes the biological action of *A. annua* (19). In addition, many previous studies have shown that *A. annua* powder or extract could enhance growth performance (20), anti-inflammation (21–23), redox status, and innate immunity in broilers and rats (24, 25). Nevertheless, the plant cytoderm restricts the release of phytochemicals inside the cytoplasm, which leads to inefficient nutritional ingredient utilization in natural herbs. Previous studies have shown that enzymatic hydrolysis technology has positive effects, such as: (i) promotion in the release and purification of biologically active substances in plant cells; (ii) shorter extraction time, less usage of solvents, and higher yield and quality of product extract than physical and chemical extraction; and (iii) improvement in absorption efficiency to the animal (26).

Our lab recently found that the dietary supplementation of enzymatically treated *Artemisia annua* L. (EA) at 1.0 g/kg greatly alleviated the oxidative stress in sows, and improved the antioxidant capacity (15). Until now, little information is available regarding the effect of EA on ameliorating the adverse effects caused by HS in sows and offspring. Therefore, we speculated that the EA supplementation in late gestation and lactation diets could ameliorate the adverse effects of HS on the lactation performance, serum hormone secretion, inflammation, and immunoglobulin transfer of multiparous sows and promote intestinal development of their preweaning offspring.

## Materials and Methods

### Preparation of EA

*Artemisia annua* was produced in China and harvested during the prosperous period. The dried whole plant (stems and leaves) of *A. annua* was pulverized and mixed thoroughly with the 0.6% compound enzymatic hydrolysate (50000 U/g cellulase: 30000 U/g pectinase = 4:1) at 55°C and a pH of 4.5 for 4 h. Following enzymatic hydrolysis, filtrate and residue were collected, dried (filtrate of enzymatic hydrolysis of *A. annua* for 3 h at 60°C; residue of enzymatic hydrolysis of *A. annua* for 5 h at 90°C), and pulverized separately, then mixed to obtain final products. The EA contained 45.02, 972.96, and 335.17 g/kg of CP (AOAC, 2005, method 991.20), DM (AOAC, 2005, method 926.12), and ether extract (AOAC, 2005, method 920.39), respectively. Total phenolic content and total flavonoid content were measured as previously described (27). In addition, the main active ingredients of EA include 1.68 ± 0.13 mg of rutin equivalents (RE)/g flavonoids, 4.77 ± 0.22 mg of gallic acid equivalents (GAE)/g phenols, 156.2 ± 0.20 mg/kg of arteannuinic acid, 62.0 ± 0.08 mg/kg of deoxyartemisinin, and 26.0 ± 0.06 mg/kg of artemisinin.

### Animals and Experimental Design

Overall, 135 sows (Large White × Landrace) of similar parity and weight on day 85 of gestation (G85) were selected and randomly distributed into 3 treatment groups with an initial 45 sows per treatment. The three treatment groups were as follows: control (Con) group, in which sows were raised in normal conditions (temperature: 27.12 ± 0.18°C, THI: 70.90 ± 0.80) and fed the basal diet. The heat-stressed (HS) and HS + EA groups, in which sows were raised in heat-stressed conditions (temperature: 30.11 ± 0.16°C, THI: 72.70 ± 0.60), and fed basal diets supplemented with 0 or 1.0 g/kg EA, respectively. The trial lasted for 50 consecutive days until day 21 of lactation (L21). The ultimate numbers of sows for analysis were 40, 41, and 42 in the Con, HS, and HS + EA groups in the present trial, respectively. The numbers of sows which were abortive, diseased (refer to metritis, lameness, poor health, etc.), deceased, and returned to estrus were 1, 1, 0, and 3 in the Con group, 0, 0, 1, and 3 in the HS group, and 1, 0, 0, and 2 in the HS + EA group, respectively.

This trial was conducted in a subtropical city in Guangdong Province in South China (subtropical climate) from July to October 2018. Comfortable control rooms contained a pad curtain with cycling water on one end of a completely closed room and large fans on the wall of the opposite end, while HS rooms were conventional rearing rooms, which are in a semi-opened building. It is worth noting that the normal adaptation temperature of sows generally should be no more than 25°C (28). In this experiment, the temperature of the Con group was higher than the normal range, although it was 3°C lower than the HS group. However, during our experiment, we did not observe any abnormal behavior of sows under 27°C, which might be due to the possibility that sows in Guangdong have partially adapted to the surrounding environment.

### Feeding and Management

The experimental basic diet is a corn-soybean diet that can meet the nutritional needs of pregnant sows and lactating sows (NRC 2012). The composition and nutritional levels of the basal diet are shown in Table 1. During the experiment, the temperature (T) and relative humidity (RH) were recorded hourly using an auto temperature and humidity recorder (W-series, Henan, China). The temperature-humidity index (THI) was calculated according to the formula of Wegner et al. (29).

**Table 1:** Ingredient composition and nutritional levels of the basic diet (g/kg, as-fed) basis).

**Items**	**Content**
Ingredients	
Corn	522.0
Soybean meal, 420 g CP/kg	240.0
Wheat bran, 157 g CP/kg	60.0
Fish meal, 640 g CP/kg	25.0
Wheat standard powder	75.0
Soybean oil	40.0
Dicalcium phosphate	12.0
Limestone	9.0
Salt	3.0
Vitamin and mineral premix^a^	3.0
Choline chloride, 500 g choline/kg	2.0
Sodium bicarbonate	2.0
Mold removal agent^c^	0.5
Mold inhibitor^d^	2.0
Carrier	4.5
Total	1,000.0
Analyzed nutritional levels	
Dry matter, g/kg	871.0
Crude protein (CP), g/kg	181.3
Ash, g/kg	57.3
Ether extract, g/kg	67.9
Neutral detergent fiber, g/kg	110.1
Acid detergent fiber, g/kg	47.8
Ca, g/kg	7.6
Total P, g/kg	6.6
Calculated nutritional levels^b^	
Digestible energy, MJ/kg	14.1
Digestible Lys, g/kg	8.6
Digestible Met + Cys, g/kg	5.1
Digestible Thr, g/kg	6.0
Digestible Trp, g/kg	1.8

a *Vitamin and mineral premix supplied per kilogram of complete diet: 50.0 mg Zn (ZnSO_4_·H_2_O), 80.0 mg Fe (FeSO_4_·H_2_O), 20.0 mg Mn (MnSO_4_·H_2_O), 5.00 mg Cu (CuSO_4_·5H_2_O), 0.14 mg I (CaI_2_O_6_), 0.30 mg Se (Na_2_SeO_3_), 13,500 IU vitamin A, 4000 IU vitamin D_3_, 90 IU vitamin E, 4 mg vitamin K_3_, 4 mg vitamin B_1_, 10 mg vitamin B_2_, 4.8 mg vitamin B_6_, 40 mg niacin, 0.034 mg vitamin B_12_, 20 mg D-pantothenate, 0.16 mg D-biotin, 2.0 mg folic acid*.

b *Calculated values according to the Chinese Feeding Standard of Swine (2004)*.

c *The main components of the mold removal agent are saccharomyces cerevisiae and sodium alginate*.

d *The main components of the mold inhibitor are propionic acid and ammonium propionate*.

From the day 85 to day 107 of gestation of the sows, every sow was housed in individual crates (2.10 × 0.6 m^2^) in the gestation facility, and the sows were allowed to drink freely and were fed twice a day (6:30 and 14:00). On day 108 of gestation, all sows were transferred into respective farrowing rooms, where all experimental settings were held constant during the gestational period. They had *ad libitum* access to their respective experimental diets, which were fed to them until weaning of their offspring on L21. One week after the piglets were born, they were assisted with creep feeding, and they were free to eat until L21. Sows were housed in individual farrowing crates (2.3 × 2.4 m^2^). Piglets were cross-fostered only within 48 h postpartum in each treatment group, and the number of litters was standardized to 10 ± 1.

### Data and Sample Collection

#### Sow and Litter Performance

Feed intakes of sows were recorded during lactation. The estrus rate of sows was recorded 7 days post-weaning of offspring. Sow backfat thickness was measured at the P_2_ position (at the last rib and 66 mm away from spine) using a digital backfat indicator (Renco Lean-Meater®, Renco Corporation, Minneapolis, USA) on G85, G114, and L21, which were used to calculate the backfat loss of lactation. Within the first 24 h after farrowing, the litter size at birth (total, live, healthy, weak, and stillborn) was recorded in this study. Moreover, individual piglet body weight (BW) as well as litter size at weaning and individual piglet BW were recorded on L21 and shown in our published research (15). These data were used to calculate the average daily gain (ADG) and survival rate of piglets during lactation in this study.

#### Serum Sample of Sows and Piglets

Blood samples (~8 mL per sow) were collected by ear venipuncture from a random subset of sows (*n* = 10 per treatment) on G85, G114, L14, and L21. Sows were fed at 06:30, the blood samples were taken 3.5 h after morning feeding (around 10:00). The sows that had blood collected at each time point were the same. On L1 and L21, six piglets were randomly selected from each treatment. At the postprandial time, blood samples were collected *via* jugular venipuncture. Blood samples were collected into sterile vacuum tubes, followed by centrifuged at 3000 × g at 4°C for 10 min. The serum was separated, transferred into micro-tubes, and then frozen immediately at −80°C for subsequent biochemical analysis.

#### Colostrum and Milk Samples of Sows

Colostrum was collected from all functional teats within 12 h postpartum; 14 day milk was collected after intramuscular injection of 30 IU oxytocin on L14. Approximately 25 mL was collected at each time period, and samples were frozen immediately at −80°C until further analysis.

#### Small Intestine Samples of Piglets

Whole gastrointestinal tracts were rapidly removed after piglets were sacrificed by exsanguination. The small intestine was peeled from the mesentery and placed on a cold stainless-steel tray. Then, segments of ~2 cm in length of the middle of the duodenum, jejunum, and ileum were immediately isolated, respectively. The intestinal tissues were gently flushed with cold phosphate-buffered saline and then placed in 10% formalin solution for morphological examination.

### Laboratory Analysis

#### Serum Hormone Profile, Endotoxin, and HSP-70 Concentrations

Serum hormone concentrations (corticosterone, insulin, glucagon, T_3_ (triiodothyronine), T_4_ (thyroxine), leptin, and prolactin), endotoxin, and heat stress protein 70 (HSP-70) were assayed as described in previous studies (30–32) using commercial radio-immunoassay kits (CUSABIO Biotech Company, Wuhan, China), then read on a spectrophotometer.

#### The Levels of Cytokines in Serum and Immunoglobulins in Colostrum and Milk

The levels of inflammatory cytokines [IL-1β (interleukin-1β), TNF-α (tumor necrosis factor-α), sCD14 (soluble cluster of differentiation 14), IL-6 (interleukin-6), IL-8 (interleukin-8), and IL-18 (interleukin-18)] in serum, and immunoglobulin G (IgG), IgA, and IgM levels in colostrum and 14 day milk of sows were analyzed as described by a previous method (33) using commercially available porcine ELISA kits (CUSABIO Biotech Company, Wuhan, China). These indices were measured with the corresponding assay kits according to the manufacturer's instructions.

Before analyzing inflammatory cytokines, lipids in colostrum and 14 day milk were removed following centrifugation at 4°C, 3000 × g for 25 min as described by Hu et al. (34). Somatic cell counts (SCC) were determined by a Fossomatic 5000 (Foss Electric, Hillerød, Denmark).

### Intestinal Morphology

The collected duodenum, jejunum, and ileum segments were dehydrated, embedded in paraffin, then stained with hematoxylin as well as eosin according to the procedures described by Song et al. (35). Villus height and crypt depth were determined using an image processing and analysis system (Leica Imaging Systems Ltd, Cambridge, UK).

### Statistical Analysis

Statistical analyses were conducted using one-way ANOVA in SPSS 17.0 software (SPSS, INC., Chicago, IL, USA), differences among groups were evaluated by Duncan's test. The individual sow and its litter were used as the experimental unit. Hormone and inflammatory cytokines in sow serum and milk were analyzed as repeated measures. The results were expressed as mean and its pooled standard errors. The significance of all data analysis was defined as *P* < 0.05.

## Results

### Lactation Performance of Sows

As shown in Table 2, higher ADG during lactation was observed in the HS + EA group compared to the HS group and Con group (*P* < 0.05). Compared with the Con group, HS decreased the average daily feed intake (ADFI); conversely, a higher ADFI was observed when the HS group was fed an EA-supplemented diet during lactation (*P* < 0.05). Sow backfat loss and the estrus rate over 7 days post-weaning and piglet survival rate did not differ among treatments (*P* > 0.05).

**Table 2:** Effects of dietary supplementation with EA on lactation performance of sows.

**Items**	**Con**	**HS**	**HS + EA**	**SEM**	***P*** **-value**
Observations of sows, *n*	40	41	42		
Sow performance					
Sow BW, kg					
G85	195.60	194.95	196.50	5.950	0.980
G114	236.50	232.95	235.20	8.650	0.780
L21	217.60	211.90	215.80	5.650	0.670
Backfat thickness, mm					
G85	18.56	19.03	18.72	0.131	0.896
G114	20.00	20.53	20.13	0.162	0.525
L21	17.69	18.19	17.78	0.170	0.614
Backfat increase during late gestation	1.44	1.50	1.41	0.193	0.326
Backfat loss during lactation	2.35	2.38	2.34	0.070	0.572
ADFI, kg	4.41^b^	4.16^c^	4.64^a^	0.051	0.001
Estrus rate over 7 days post-weaning, %	91.12	94.44	91.11	1.901	0.572
Litter performance					
Litter size at birth, total	11.36	10.86	11.03	0.161	0.963
Litter size at birth, live	9.64	10.13	10.38	0.150	0.368
Litter size at birth, healthy	9.44	9.95	10.27	0.161	0.266
Litter size at birth, weak	0.18	0.18	0.11	0.030	0.276
Litter size at birth, stillborn	0.66	0.62	0.64	0.090	0.491
Piglet ADG, g/day	198.09^b^	187.90^c^	210.15^a^	2.450	0.037
Piglet survival rate, %	90.90	85.76	87.96	1.030	0.882

*Different superscript letters (a, b, c) indicate that there are significant differences between the groups (P < 0.05)*.

*EA, enzymatically treated Artemisia annua L.; Con, control group; HS, heat stress group*.

### Hormone, Endotoxin, and HSP-70 Levels in Serum of Sows

As shown in Tables 3, 4, compared with the Con group, sows in the HS group had lower levels of T_3_ (L14, L21) and higher levels of endotoxin (G114, L14) and HSP-70 (G114) (*P* < 0.05). In contrast, dietary EA supplementation resulted in higher levels of T_3_ (L14, L21) and lower concentrations of endotoxin (G114, L14) and HSP-70 (G114) compared with the Con and HS groups (*P* < 0.05).

**Table 3:** Effects of dietary EA supplementation on hormone levels in the serum of sows.

**Items**	**Con**	**HS**	**HS + EA**	**SEM**	***P*** **-value**
Corticosterone, ng/mL					
G85	195.83	189.71	189.70	3.960	0.778
G114	189.71	208.06	146.89	15.331	0.539
L14	342.63	367.11	330.40	19.170	0.378
L21	330.40	329.23	330.40	32.041	0.887
Insulin, mIU/L					
G85	117.50	78.67	85.64	16.120	0.771
G114	34.42	42.21	39.37	2.270	0.714
L14	34.54	54.92	33.42	26.480	0.895
L21	57.54	42.08	57.36	39.754	0.684
Glucagon, ng/L					
G85	1406.47	1437.95	1399.73	70.855	0.755
G114	1507.23	1364.85	1355.20	59.940	0.443
L14	1390.72	1362.67	1412.22	57.300	0.514
L21	1306.90	1391.69	1305.42	70.552	0.372
T_3_, ng/mL					
G85	3.70	3.55	3.60	0.230	0.693
G114	7.23	6.98	7.56	0.111	0.205
L14	2.54^b^	2.34^c^	2.99^a^	0.136	0.004
L21	3.50^b^	3.00^c^	5.65^a^	0.440	0.001
T_4_, ng/mL					
G85	41.25	37.13	40.80	3.070	0.838
G114	41.03	42.78	37.80	2.950	0.615
L14	46.43	47.55	41.99	3.610	0.254
L21	42.75	50.63	48.79	7.560	0.881
Leptin, ng/mL					
G85	12.85	13.74	11.07	0.630	0.314
G114	11.28	12.70	11.42	0.470	0.403
L14	12.36	11.95	12.38	0.360	0.863
L21	13.09	11.08	10.46	0.600	0.518
Prolactin, ng/mL					
G85	770.38	571.43	811.23	129.412	0.881
G114	1166.07	1008.45	1079.18	35.440	0.170
L14	703.31	795.50	898.69	61.220	0.324
L21	1149.08	1057.07	1141.47	17.121	0.228

*The superscript letters (a, b, c) indicate that there are significant differences between the groups (P < 0.05). T_3_, triiodothyronine; T_4_, tetraiodothyronine; G85, day 85 of gestation; G114, day 114 of gestation; L14, day 14 of lactation; L21, day 21 of lactation*.

**Table 4:** Effects of dietary supplementation with EA on endotoxin and HSP-70 levels in the serum of sows.

**Items**	**Con**	**HS**	**HS + EA**	**SEM**	***P*** **-value**
Endotoxin, EU/L					
G85	455.94	470.84	368.40	39.850	0.426
G114	515.78^b^	576.25^a^	508.84^c^	26.130	0.026
L14	508.28^b^	516.33^a^	326.16^c^	42.371	0.045
L21	369.07	392.41	282.96	45.121	0.131
HSP-70, ng/mL					
G85	2.32	2.52	2.30	0.110	0.440
G114	4.40^b^	4.60^a^	2.16^c^	0.530	0.038
L14	2.87	3.06	2.54	0.641	0.266
L21	1.09	1.23	1.12	0.243	0.523

*The superscript letters (a, b, c) indicate that there are significant differences between the groups (P < 0.05). HSP-70, heat shock protein 70*.

### Inflammatory Cytokines Levels in Serum, Colostrum, and 14 Day Milk

Compared with the Con group, the HS group had higher levels of TNF-α (G114) and IL-6 (G114, L14) in the serum of sows, as well as TNF-α (L1) and IL-6 (L1) levels in the serum of piglets (*P* < 0.05). Dietary EA supplementation resulted in lower levels of TNF-α and IL-6 levels compared with the Con and HS groups in the serum of sows and their offspring (*P* < 0.05; Tables 5, 6). Compared with the Con group, the HS group had higher levels of TNF-α, IL-6, and SCC in the colostrum of sows (*P* < 0.05). Dietary EA supplementation resulted in lower levels of TNF-α, IL-6, and SCC in the colostrum compared with the Con and HS groups (*P* < 0.05; Table 7).

**Table 5:** Effects of dietary supplementation with EA on the inflammatory cytokine levels in the serum of sows.

**Items**	**Con**	**HS**	**HS + EA**	**SEM**	***P*** **-value**
TNF-α, ng/mL					
G85	1.29	1.25	1.24	0.110	0.835
G114	1.71^b^	2.28^a^	1.23^c^	0.260	0.045
L14	0.82	0.87	0.76	0.100	0.728
L21	0.61	0.68	0.60	0.080	0.895
IL-1β, pg/mL					
G85	185.77	181.33	172.25	6.080	0.342
G114	129.55	143.10	126.40	1.080	0.907
L14	116.21	159.15	120.29	1.740	0.316
L21	95.34	83.86	110.87	6.614	0.622
sCD14, ng/mL					
G85	7.68	7.25	7.76	0.155	0.943
G114	3.23	3.49	4.02	0.520	0.686
L14	2.47	2.49	2.08	0.480	0.665
L21	1.65	1.75	1.63	0.113	0.885
IL-6, pg/mL					
G85	63.59	62.65	62.12	0.361	0.954
G114	31.01^b^	43.37^a^	26.66^c^	0.422	0.025
L14	10.76^b^	15.25^a^	8.31^c^	0.162	0.049
L21	9.46	9.16	8.91	0.132	0.754
IL-8, pg/mL					
G85	1010.85	1093.28	1094.37	98.320	0.987
G114	1145.96	1098.16	931.07	65.411	0.371
L14	823.51	872.25	892.46	75.762	0.985
L21	724.02	739.50	720.56	91.960	0.979
IL-18, pg/mL					
G85	1580.38	1453.33	1448.69	81.903	0.666
G114	601.26	611.50	679.36	64.150	0.665
L14	413.04	441.62	339.27	50.160	0.506
L21	404.08	393.35	391.14	57.980	0.898

*The superscript letters (a, b, c) indicate that there are significant differences between the groups (P < 0.05). TNF-α, tumor necrosis factor-α; IL-1β, interleukin-1β; sCD14, soluble cluster of differentiation 14; IL-6, interleukin-6; IL-8, interleukin-8; IL-18, interleukin-18*.

**Table 6:** Effects of maternal supplementation with EA on the inflammatory cytokine levels in the serum of piglets.

**Items**	**Con**	**HS**	**HS + EA**	**SEM**	***P*** **-value**
L1					
TNF-α, ng/mL	0.04^b^	0.08^a^	0.03^c^	0.001	0.049
IL-1β, pg/mL	7.25	8.52	6.68	0.560	0.420
sCD14, ng/mL	0.062	0.074	0.073	0.001	0.390
IL-6, pg/mL	3.53^b^	4.57^a^	2.15^c^	0.061	0.037
IL-8, pg/mL	229.75	269.81	223.29	13.920	0.350
IL-18, pg/mL	115.28	129.49	102.46	7.980	0.379
L21					
TNF-α, ng/mL	0.03	0.04	0.04	0.001	0.598
IL-1β, pg/mL	6.75	6.65	6.83	0.040	0.318
sCD14, ng/mL	0.079	0.086	0.082	0.002	0.260
IL-6, pg/mL	1.64	1.61	1.65	0.150	0.996
IL-8, pg/mL	276.43	300.99	264.83	26.981	0.855
IL-18, pg/mL	137.92	154.23	138.65	19.363	0.932

*The superscript letters (a, b, c) indicate that there are significant differences between the groups (P < 0.05). TNF-α, tumor necrosis factor-α; IL-1β, interleukin-1β; sCD14, soluble cluster of differentiation 14; IL-6, interleukin-6; IL-8, interleukin-8; IL-18, interleukin-18; L1, day 1 of lactation*.

**Table 7:** Effects of dietary supplementation with EA on the inflammatory cytokine and SCC levels in the colostrum and 14 day milk of sows.

**Items**	**Con**	**HS**	**HS + EA**	**SEM**	***P*** **-value**
Colostrum					
TNF-α, ng/mL	0.25^b^	0.43^a^	0.20^c^	0.021	0.043
IL-1β, pg/mL	31.03	32.51	32.55	2.610	0.809
sCD14, ng/mL	0.44	0.61	0.50	0.041	0.753
IL-6, pg/mL	6.64^b^	9.72^a^	5.70^c^	0.950	0.045
IL-8, pg/mL	893.96	937.49	893.10	45.920	0.917
IL-18, pg/mL	426.57	412.03	422.00	27.001	0.765
SCC, 10^6^/mL	4.98^b^	5.34^a^	4.86^c^	0.030	0.046
14 day milk					
TNF-α, ng/mL	0.08	0.07	0.10	0.004	0.505
IL-1β, pg/mL	30.67	34.03	36.81	4.840	0.695
sCD14, ng/mL	0.02	0.04	0.04	0.006	0.206
IL-6, pg/mL	3.42	2.32	3.94	0.280	0.933
IL-8, pg/mL	1061.65	913.31	1018.68	176.810	0.929
IL-18, pg/mL	204.64	261.01	335.39	30.180	0.425
SCC, 10^6^/mL	3.78	3.81	3.63	0.040	0.109

*The superscript letters (a, b, c) indicate that there are significant differences between the groups (P < 0.05). TNF-α, tumor necrosis factor-α; IL-1β, interleukin-1β; sCD14, soluble cluster of differentiation 14; IL-6, interleukin-6; IL-8, interleukin-8; IL-18, interleukin-18; SCC, somatic cell count*.

### Immunoglobulin Levels in Colostrum, 14 Day Milk of Sows, and Serum of Nursing Piglets

As shown in Tables 8, 9. There were no treatment effects on IgA, IgG, and IgM levels in the colostrum, 14 day milk of sows, and serum of nursing piglets (*P* > 0.05).

**Table 8:** Effects of dietary supplementation with EA on immunoglobulin levels in the colostrum and 14 day milk of sows.

**Items**	**Con**	**HS**	**HS + EA**	**SEM**	***P*** **-value**
Colostrum, mg/mL					
IgA	8.33	8.69	8.88	1.051	0.970
IgG	46.90	45.70	47.55	2.750	0.988
IgM	0.23	0.16	0.18	0.011	0.795
14 day milk, mg/mL					
IgA	1.65	1.49	1.44	0.050	0.519
IgG	0.68	0.99	0.87	0.070	0.405
IgM	0.021	0.021	0.019	0.001	0.486

*IgA, immunoglobulin A*.

**Table 9:** Effects of maternal dietary supplementation with EA on immunoglobulin levels in the serum of nursing piglets.

**Items**	**Con**	**HS**	**HS + EA**	**SEM**	***P*** **-value**
L1, μg/mL					
IgA	10.20	10.61	10.22	0.581	0.954
IgG	58.24	57.11	56.22	0.602	0.412
IgM	3.37	3.56	3.49	0.071	0.627
L21, μg/mL					
IgA	12.71	12.53	12.81	0.160	0.796
IgG	58.45	59.47	58.22	0.261	0.112
IgM	3.70	3.79	3.83	0.060	0.629

*IgA, immunoglobulin A; L1, day 1 of lactation*.

### Intestinal Morphology of Piglets

As shown in Table 10. Compared with the Con group, the villus height decreased in the duodenum at birth (L1) and in the ileum at weaning (L21) (*P* < 0.05) in the HS group. Compared with the Con and HS groups, EA supplementation increased the villus height in the duodenum at birth and in the ileum at weaning (*P* < 0.05).

**Table 10:** Effects of maternal dietary supplementation with EA on small intestinal morphology of nursing piglets.

**Items**	**Control**	**HS**	**HS + EA**	**SEM**	***P*** **-value**
Duodenum					
L1					
Villus height, μm	490.15^b^	462.33^c^	584.33^a^	27.350	0.045
Crypt depth, μm	65.55	59.04	76.52	5.980	0.321
Villus/crypt	7.47	7.86	7.64	0.760	0.821
L21					
Villus height, μm	288.38	315.91	293.89	12.700	0.461
Crypt depth, μm	142.79	132.08	148.42	6.780	0.412
Villus/crypt	2.07	2.45	1.99	0.151	0.274
Jejunum					
L1					
Villus height, μm	462.90	496.53	497.85	47.150	0.806
Crypt depth, μm	55.80	60.01	62.21	3.791	0.371
Villus/crypt	8.30	8.26	8.01	0.510	0.592
L21					
Villus height, μm	300.33	283.43	306.26	10.250	0.466
Crypt depth, μm	132.20	138.76	123.11	8.020	0.526
Villus/crypt	2.27	2.05	2.49	0.200	0.138
Ileum					
L1					
Villus height, μm	463.37	498.41	532.63	40.730	0.826
Crypt depth, μm	48.44	46.87	50.10	1.090	0.890
Villus/crypt	9.56	10.62	10.63	1.070	0.891
L21					
Villus height, μm	322.93^b^	308.17^c^	378.63^a^	13.710	0.048
Crypt depth, μm	91.21	82.71	101.60	5.310	0.600
Villus/crypt	3.54	3.72	3.73	0.150	0.249

*The superscript letters (a, b, c) indicate that there are significant differences between the groups (P < 0.05). L1, day 1 of lactation; L21, day 21 of lactation*.

## Discussion

HS exposure in later pregnancy induces oxidative stress (36), reduces digestive enzyme activities and immunity capability (37), causes inflammation and intestinal damage (38), and leads to an insufficient nutrient supply for sows, which have a series of detrimental effects on the reproductive and lactation performance as well as health condition of sows (39). Therefore, it is particularly important to find a suitable method to alleviate the adverse effects of HS on sows and their offspring.

When the ambient temperature exceeds the sow's critical temperature for evaporation, the sow is under HS (40). HS reduced the appetite and feed intake which impairs the compensatory capacity of sows (41) and sows usually consume less feed in order to reduce metabolic heat production (42). Disrupted physiological balance and reduced milk production and feed conversion efficiency of sows under HS have also been observed (43). To date, many previous studies have shown that diets supplemented with *A. annua* extract or *A. annua* leaf powder increased ADG and ADFI (14, 23, 44) and alleviated HS-induced body weight reduction and intestinal morphology impairment in broilers (19). Beyond this, emerging evidence also demonstrated that a diet directly supplemented with EA increased ADG and nutrient digestibility in weaned pigs (45, 46). Similar to this trial, the higher ADG in weaned piglets and ADFI in sows fed EA-supplemented diets was observed during lactation compared with the Con and HS groups. The crude protein, essential amino acids, minerals, vitamins, and flavonoids in EA delivered through the colostrum are important for growth and development of piglets, which is probably the reason for the enhancement of ADG (14, 45). The higher ADFI in the sows fed with supplemental EA might be due to the fact that artemisinin and phenolic compounds, including curcumin, resveratrol, and gallate could arouse appetite and reduce the heat from immuno-inflammatory responses.

A balanced physiological state is key for animal health. HS causes endocrine hormone increases in sows during pregnancy and lactation (3). T_3_ is mainly derived by deiodination of T_4_ which is known to stimulate lactation. HS could induce decreases in deiodinase activity to decrease T_3_ expression (47). In addition, published experimental research indicated that HS negatively affected thyroid status, including depression in pituitary-thyroid axis activity and peripheral metabolism of T_3_ and T_4_ through LPS-induced pro-inflammatory stimulus (48). In addition, HS injures the barrier of the intestine and induces the increment of endotoxins (i.e., LPS) from bacteria flow across the mesenteric-drained viscera. The transfer of endotoxins to the interior circulation contributes to systemic inflammation (49). A high content of HSP-70 is widely considered as a pointer of various environmental stress responses such as HS (50). Many previous studies have shown that HS reduced T_3_ concentration (51) and activity and increased endotoxin (33) and HSP-70 levels (4) in the blood of broilers and sows. In this study, HS reduced the T_3_ level in the serum of sows. Meanwhile, HS significantly increased the expressions of endotoxin and HSP-70 in the serum, which indicated that the HS model was established in sows in the current study. Our results were consistent with the findings reported in previous studies (4, 51). Whereas, EA supplementation effectively increased the T_3_ level and reduced the endotoxin level, which might be due to the fact that artemisinin and arteannuinic acid have antioxidant activity (24), bactericidal and antibacterial functions (52), and improve deiodinase activity. Thus, EA directly or indirectly alleviated the inflammatory response triggered by HS. The downregulation of HSP-70 level in heat-stressed sows following supplemental EA might be associated with the modulation of reduced endotoxin-induced pro-inflammatory cytokine expression in the present study.

The gastrointestinal tract is predominantly responsive to HS which destroys the intestinal mucosal structure (53). Subsequently, pathogens and toxins enter the damaged mucosal barrier, which stimulates the immune inflammatory response and leads to the flow of inflammatory cytokines into the colostrum and milk *via* blood circulation (54). The levels of inflammatory cytokines (such as IL-1β, IL-6, and TNF-α) in blood are considered to be an indicator to assess inflammation (23). HS upregulates IL-1β, IL-6, and TNF-α levels in the blood of pigs (55), which manifests as a serious proinflammatory stimulus. Song et al. (4, 56) reported that EA reduced proinflammatory cytokines and restored intestinal mucosal integrity caused by HS in broilers. Niu et al. (45) also showed that EA decreased the concentrations of IL-1β, IL-6, and TNF-α in the small intestine of weaned pigs. Accumulating evidence suggested that artemisinin and phenolic compounds (such as gallate, curcumin, resveratrol, and chlorogenic acids) in EA could exhibit a greater ability to prohibit the inflammatory response induced by lipopolysaccharide (LPS) (21, 57). Our recent studies have shown that EA extractive artemisinin inhibited LPS-induced activation of the mitogen-activated protein kinase (MAPK) and nuclear factor-κB (NF-κB) signaling pathways in porcine mammary epithelial cells (58). Our findings were consistent with the findings of previous studies (21, 56–58). The mechanism has been observed that sesquiterpenes (artemisinin and arteannuinic acid) as well as flavonoids (casticin and chrysosplenol D) from EA can attenuate the NF-κB/ nod-like receptor family pyrin domain containing 3 (NLRP3) pathways as well as toll-like receptor 4 (TLR4)/NF-κB and MAPK signaling pathways to further inhibit the expression of various inflammatory cytokines genes, including TNF-α and IL-1β (59, 60). In the present study, the addition of EA in sow diets effectively reduced the increase in inflammatory cytokine levels in the serum, colostrum, and milk of sows and piglets and further relieved the inflammation caused by HS. Hence, we speculated that EA attenuated the LPS-induced inflammatory pathways in the gut and mammary glands to alleviate inflammation in heat-stressed sows. Furthermore, the reduction resulting from EA in the colostrum might indicate the lower incidence of LPS-induced mastitis in sows during lactation (58).

The small intestine is not only the main place for the body to digest and absorb nutrients, but also the largest immune organ of the body (54). Oxidative and inflammatory stress brought by HS damages the intestinal villus structure, reduces villus height (33), increases intestinal permeability, and subsequently results in diarrhea and growth retardation (61). Small intestine mucosal villus height and crypt depth are important indicators for evaluating intestinal development (46). Intestinal villus height is positively correlated with the surface area in contact with the chyme, which is an indicator of the intestinal absorption capacity (62). Studies have shown that HS affected the structural integrity and barrier function of piglet intestines through maternal effect directly and colostrum intake indirectly (10, 61). In the current study, we investigated the vertically transmitted effect of maternal effect on offspring's small intestinal health. Maternal supplementation with 1.0 g/kg of EA effectively alleviated the damage of HS to the small intestine of offspring by increasing intestinal villus height. However, Niu et al. (46) reported that diet supplemented with 2.0 g/kg of EA (but not 1.0 g/kg of EA) decreased crypt depth and increased villus height and villus height to crypt depth ratio in both the jejunum and ileum of piglets. The different results between these two studies indicates the optimal dose of EA for weaning piglets and sows is controversial. Different from the direct effect of EA on the gut health of sows, EA could indirectly protect mucosal health in piglets by means of reducing oxidative and inflammatory stimulus caused by milk from sows (15). The above results suggested that the decreased oxidative and inflammatory stress in the colostrum and milk in this trial might be the reason why EA protected gut health in piglets.

## Conclusion

**Figure 1:**
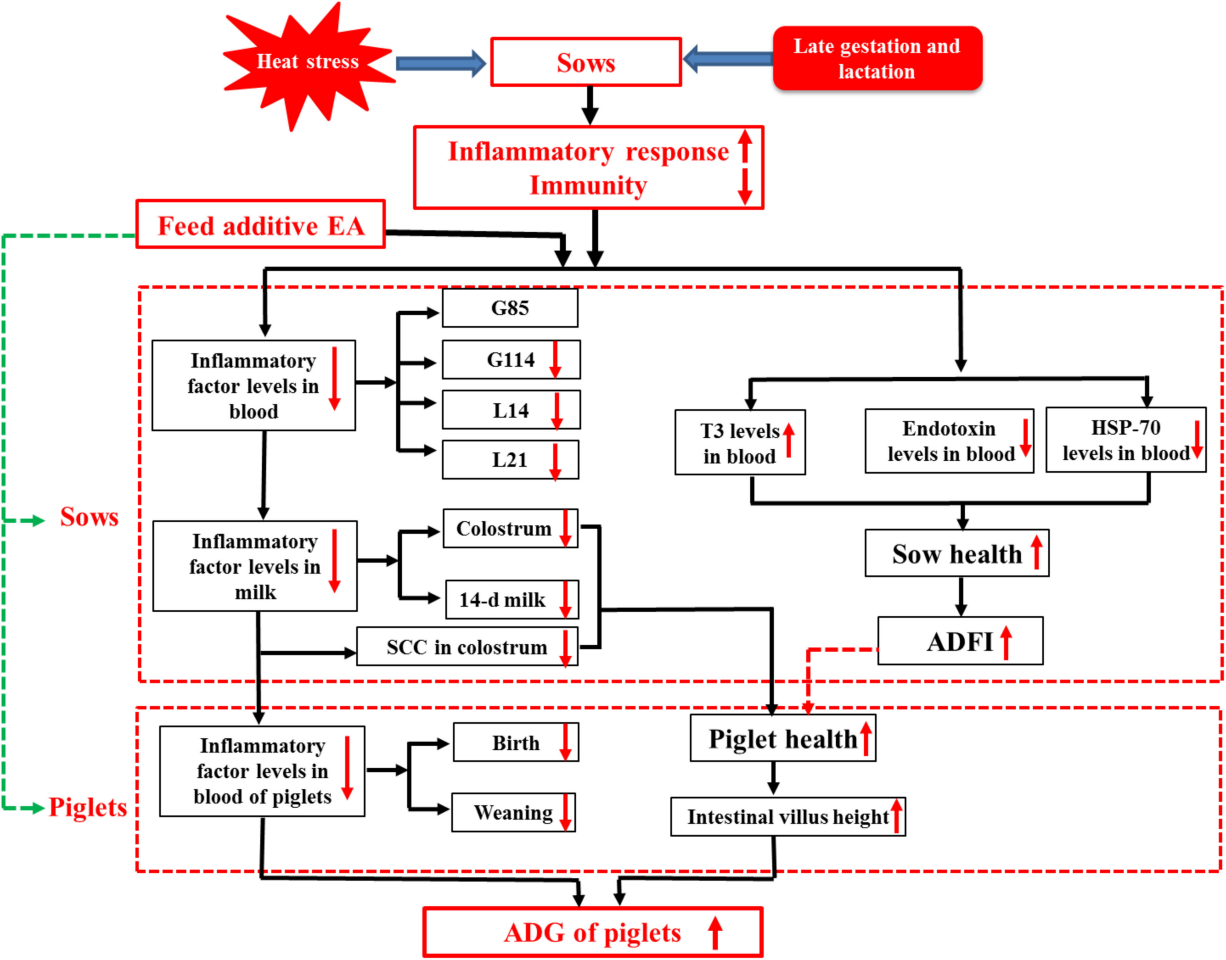
Dietary enzymatically treated *Artemisia annua* L. (EA) supplementation at 1.0 g/kg during late gestation and lactation increased ADFI, decreased endotoxin and inflammatory factor levels in the serum and colostrum of heat-stressed sows during lactation, and increased ADG and improved the gut development of their offspring.

Our present study demonstrated for the first time that dietary supplementation with enzymatically treated *Artemisia annua* L. at 1.0 g/kg exhibited beneficial effects on the lactation performance, serum hormone level, and inflammatory response of sows under HS and the gut health of their offspring (Figure 1). The results of this study provided a new perspective for alleviating the inflammatory response of sows, and at the same time supplied a new nutritional management strategy for alleviating the HS response in animal production.

## Data Availability Statement

The original contributions presented in the study are included in the article/supplementary material, further inquiries can be directed to the corresponding authors.

## Ethics Statement

The animal study was reviewed and approved by South China Agricultural University Animal Care and Use Committee (Guangzhou, China) (SCAU-AEC-2010-0416).

## Author Contributions

LX, WZ, FC, SZ, and WG designed the experiments. WZ, LX, HZ, and ZT performed the experiments. WZ analyzed the data and wrote the manuscript. FC, MR, WG, and SZ conducted the final proofreading. All authors have read and agreed to the published version of the manuscript.

## Funding

This study was financially supported by the National Key R&D Program of China (No. 2021YFD1300700), the National Natural Science Foundation of the P. R. of China (Nos. 31872364 and 31802067), Guangdong Basic and Applied Basic Research Foundation (No. 2021A1515010440), Science and Technology Program of Guangzhou (No. 202102020056), and Major Science and Technology Projects in Anhui Province (201903a06020002).

## Conflict of Interest

The authors declare that the research was conducted in the absence of any commercial or financial relationships that could be construed as a potential conflict of interest.

## Publisher's Note

All claims expressed in this article are solely those of the authors and do not necessarily represent those of their affiliated organizations, or those of the publisher, the editors and the reviewers. Any product that may be evaluated in this article, or claim that may be made by its manufacturer, is not guaranteed or endorsed by the publisher.
